# Disease burden and direct medical costs of incident adult ADHD: A retrospective longitudinal analysis based on German statutory health insurance claims data

**DOI:** 10.1192/j.eurpsy.2020.84

**Published:** 2020-10-01

**Authors:** Berit Libutzki, Melanie May, Markus Gleitz, Michael Karus, Benno Neukirch, Catharina A. Hartman, Andreas Reif

**Affiliations:** 1 Department of Psychiatry, Interdisciplinary Center Psychopathology and Emotion regulation (ICPE), University of Groningen, University Medical Center Groningen, Groningen, The Netherlands; 2 HGC Healthcare Consultants GmbH, Düsseldorf, Germany; 3 MEDICE Arzneimittel Pütter GmbH & Co KG, Iserlohn, Germany; 4 Hochschule Niederrhein, University of Applied Sciences, Krefeld, Germany; 5 Department of Psychiatry, Psychosomatic Medicine and Psychotherapy, University Hospital Frankfurt, Frankfurt am Main, Germany

**Keywords:** Adult ADHD (aADHD), claims data, comorbidities, healthcare costs, medication

## Abstract

**Background.:**

Adult attention-deficit/hyperactivity disorder (aADHD) is still a largely unrecognized psychiatric condition despite its strong impact on individuals’ well-being. Here, we describe the healthcare situation of individuals with incident aADHD over 4 years before and 4 years after initial administrative diagnosis.

**Methods.:**

A retrospective, longitudinal cohort analysis was conducted using German claims data. The InGef database contained approximately 5 million member-records from over 60 nationwide statutory health insurances (SHI). Individuals were indexed upon initial diagnosis of aADHD.

**Results.:**

Average age at diagnosis of aADHD was 35 years, and 60% of individuals were male. Comorbidities, resource use, and healthcare costs were substantial before initial diagnosis and decreased within the 4 years thereafter. Only 32% of individuals received initial ADHD medication and adherence was low. The majority received psychotherapy. Individuals with initial ADHD medication showed the highest share in comorbidities, physician visits, medication use for comorbidities, psychotherapy, and costs. Overall, healthcare costs were at over €4,000 per individual within the year of aADHD diagnosis.

**Conclusions.:**

We conclude that earlier recognition of aADHD could prevent the development and aggravation of comorbid mental illnesses. At the same time, comorbid conditions may have masked (“over-shadowed”) aADHD and delayed diagnosis. The burden of disease in aADHD is high, which was noticeable especially among individuals who received initial ADHD-medication, suggesting that psychopharmacological treatment was mainly considered for the most severely ill. We conclude that measures to facilitate access of aADHD patients to clinical experts are required to improve reality of care in the outpatient setting.

## Introduction

Attention-deficit/hyperactivity disorder (ADHD) is a common neurodevelopmental disorder that often persists into adulthood and is then termed “adult ADHD” (aADHD). Over the last years, aADHD has been increasingly recognized as an impairing mental health condition [1]. A timely recognition of aADHD and initiation of treatment may, however, be compromised by a lack of consensus among physicians regarding core aADHD symptoms and diagnostic procedures [2]. Accordingly, recent studies have shown that aADHD is still underdiagnosed as is evident from the low administrative prevalence of aADHD in Germany (between 0.04% in 2007 [3] and 0.66% in 2019 [4–6]), compared to the population prevalence ranging between 1 and 7% in high-income countries [7].

aADHD is a risk factor for various mental disorders [6,8,9]. The frequent occurrence of comorbidities (“diagnostic over-shadowing”) is a factor that may lead to misdiagnoses or delayed diagnosis, and in turn delay treatment. A recent study based on statutory health insurance (SHI) claims data has shown that adults with ADHD show significantly higher medical costs than those without, due to ADHD and associated comorbidities [6]. It is possible that early detection and treatment initiation of aADHD reduce the burden of illness and improve the quality of life of those affected, because the development of comorbidities may be prevented [10–12]. The resulting decrease in healthcare resource use and avoided comorbidities may lead to long-term cost savings.

Research on the impact of aADHD diagnosis, therapies, and the development of costs is, therefore, urgently needed. The aim of this study was to describe the healthcare situation of individuals with incident aADHD, based on German SHI claims data. Our longitudinal study analyzed the trajectory of aADHD 4 years before and after diagnosis.

## Method

### Study design and participants

#### SHI claims data study

The analysis was conducted using German claims data from the InGef research database, containing approximately 5 million anonymized member-records from over 60 nationwide SHIs. The sample is representative of the German population in terms of age and sex, and it is widely used for real-world evaluation. There is a good overall accordance of the database with the German population in terms of morbidity, mortality, and drug usage [13].

**Figure 1.**
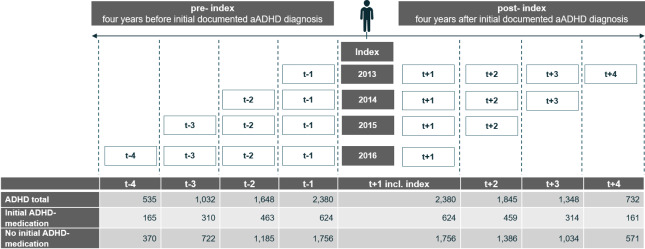
Indexing of individuals with ADHD in yearly cohorts.

The study was designed to capture individuals with incident aADHD, hence individuals who receive an initial diagnosis of ADHD as adults and who have not yet received treatment. The study was conducted as a longitudinal cohort design study. Data were available from 2012 to 2017. Individuals with aADHD were identified between 2013 and 2017 upon documented diagnosis of ICD-10 GM F90, including F90.0, F90.1, F90.8, F90.9, as a confirmed outpatient or inpatient (main or secondary) diagnosis. Annual cohorts were built to allow a maximum observation period of 4 years before (*t* − 4, *t* − 3, *t* − 2, *t* − 1) and after diagnosis (*t* + 1, *t* + 2, *t* + 3, *t* + 4) with each year of observation as 365 days, Figure 1.

The first year of follow-up (*t* + 1) comprises the quarter of index, in which the initial aADHD diagnosis was documented, and the subsequent three-quarters. Three exclusion criteria were applied. First, individuals had to be continuously insured throughout 2012–2017 to avoid loss to follow-up, also excluding 12 individuals who died during observation. Second, to ensure incident aADHD, no documented ADHD diagnosis or prescription of ADHD-specific medication was allowed at minimum 365 days before index (cohort 2013) to a maximum of 1,460 days before index (cohort 2016). Third, individuals had to be between 18 and 55 years of age; for example, an individual with index in 2013 had to be at least 19 years and at maximum 51 years at index. Incident adults above 55 years of age were excluded to prevent bias in older age groups due to the overall high morbidity after this age. Applying these criteria, 2,380 individuals with incident aADHD out of 3,129,423 continuously insured individuals were eligible.

The aADHD population was stratified according to the prescription of ADHD-specific medication within *t* + 1 Figure 1. Individuals with at least two prescriptions within *t* + 1 were classified as “initial ADHD medication,” while individuals with no or only one prescription were classified as “no initial ADHD medication.” Here, we considered the issuing of at least two prescriptions as an indication for initiation of a specific, longer-term ADHD medication treatment. Individuals were kept in their assigned subgroup irrespective of subsequent ADHD medication status. A maximum of 5% of those assigned to “no initial ADHD medication” in *t* + 1 received more than one prescription in *t* + 2, *t* + 3, and *t* + 4.

### Outcomes and statistical analyses

Basic demographic information (age, sex) was extracted for all identified individuals with aADHD. Outcomes measured were: speciality of the diagnosing physician, physician visits during observation; speciality of the initial prescriber of ADHD-specific medication; predefined comorbidities commonly associated with aADHD; prescription of disorder-specific medication, psychotherapy, hospitalization, healthcare costs, sickness benefits, and sick leave days. In Germany, sickness benefits funded by the SHI are available after more than 6 weeks of continuous inability to work. Prior to that the employer continues salary payment, which is not observable within this dataset. The amount of sickness benefits is calculated based on the regular income. Sick leave days are defined as all days a person is not able to work based on doctor’s note. Sick leave days are documented independently of sickness benefits.

Office-based physicians were classified according to their medical speciality based on AGS (physician speciality code): “GP” (general practitioner) AGS 1, 2, 3. “Psych MD” was used for physicians practicing as psychiatrists and medical psychotherapists (AGS 47, 51, 53, 58, 60, 61). “Psych” was used for psychological psychotherapists (AGS 68, 69). Psychotherapy (doctor’s fee scale [“EBM”] chapter 35) and up to five probatory sessions before long-term psychotherapy (EBM 35150, 30931) were assessed. The following psychiatric comorbidities were analyzed via ICD-10 GM: substance use disorders (SUD) (F10–F19); mood disorders (F30–F39); anxiety disorders (F40–F48); personality and behavioral disorders (F60–F69) and all mental and behavioral disorders (F00–F99). Medication was documented when handed in at the pharmacy based on the anatomical-therapeutic-chemical classification system (ATC). ADHD-specific medication: N06BA02 dexamphetamine, N06BA04 methylphenidate, N06BA09 atomoxetine, N06BA12 lisdexamfetamine [14]; treatment of psychiatric comorbidities: N05A antipsychotics, N05B anxiolytics, N05C hypnotics and sedatives, N06A antidepressants, N07BB alcohol dependence, N07BC opiate dependence. Note: inpatient ADHD-specific medication was not visible within the database.

Healthcare costs (all-cause) were documented as follows: healthcare costs total, inpatient care, outpatient care, psychotherapy, medical aids and remedies, medication, and sickness benefits. To adjust for high-cost cases, costs of individuals with healthcare costs above the 95th percentile were replaced (winsorized) by the upper cost limit of the 95th percentile. No individual was excluded.

This study presents descriptive information of individuals with aADHD 4 years before and 4 years after initial diagnosis on the basis of yearly cohorts indexed in 2013, 2014, 2015, and 2016, with individual observation time pre-index and post-index, see Figure 1. Tests for significance were conducted within the cohorts indexed in the year 2014 and 2015, comparing *t* − 2 and *t* + 2 to *t* + 1 for time-based comparisons; subgroup comparisons were done for *t* + 1. Significance for comorbidities and therapies was tested using Chi-square test. Outcomes tested were: mental and behavioral disorders (any), mood disorders, prescription of antidepressants, ADHD-specific medication, hospitalization due to psychiatric primary indication, and psychotherapy. In addition, odds ratios (OR) and 95% upper and lower confidence intervals (95% CI) were calculated if applicable. For cost comparisons, the Wilcoxon test was used. Outcomes tested were: total healthcare costs and psychotherapy costs. In addition, the statistical difference in age and sex between the group of individuals with ADHD-medication and without was tested using Student’s *t* test and Chi-square test, respectively. For continuous variables reporting means, standard deviation (SD) was given. For data storage and processing, Microsoft Office Excel^®^ 2010 (Microsoft Corporation, WA) and R (Version Microsoft R Open 3.5.0) were used.

#### Ethical approval and data protection

The analysis did not involve any decisions regarding interventions or the omission of interventions. Accordingly, institutional review board/ethical approval and informed consent of the individual were not required. Moreover, all individual patient data are de-identified in the research database to comply with German data protection regulations. Patient numbers below five were not reported.

## Results

### Demographic and clinical characteristics of the patient cohort

A study cohort of 2,380 individuals with incident aADHD was identified, 60% being male and, on average, 35 years old at initial aADHD diagnosis, Table 1. Age and sex were not statistically significant between individuals with initial ADHD medication and without. Most individuals received diagnosis from a specialist: 23% in the inpatient setting and another 33% by the outpatient specialist (Psych MD); 41% were diagnosed by the GP. The majority of individuals with initial ADHD medication were diagnosed by outpatient specialists (56%: 352 out of 624), whereas almost half of the individuals without initial ADHD medication were diagnosed by GPs (46%: 807 out of 1,756).

**Table 1.** Demography, diagnosing physician, and prescriber of ADHD-specific medication.

Age and sex^a^	ADHD total	Initial ADHD medication	No initial ADHD medication
Total, *n* (%)	2,380 (100%)	624 (26%)	1,756 (74%)
Female, *n* (%)	961 (100%)	267 (28%)	694 (72%)
Male, *n* (%)	1,419 (100%)	357 (25%)	1,062 (75%)
Age (years) mean ± SD	34.6 ± 9.9	34.9 ± 9.3	34.6 ± 10.2
Age difference initial ADHD medication vs. no medication		*p* = 0.42	
Sex difference initial ADHD medication vs. no medication		*p* = 0.17	
Diagnosing physician (speciality of office-based outpatient) and inpatient diagnosis of initial aADHD diagnosis at index quarter (no significance tested)			
General practitioner	980 of 2,380 (41%)	173 of 624 (28%)	807 of 1,756 (46%)
Psych MD	778 of 2,380 (33%)	352 of 624 (56%)	426 of 1,756 (24%)
Psych	95 of 2,380 (4%)	17 of 624 (3%)	78 of 1,756 (4%)
Inpatient diagnosis	557 of 2,380 (23%)	178 of 624 (29%)	379 of 1,756 (22%)
Number of individuals with at least one prescription of ADHD-specific medication in the first year of follow-up			
At least one prescription of ADHD-specific medication	759 of 2,380 (32%)	624 of 624 (100%)	–
Prescribing physician of ADHD-specific medication (outpatient) for those with at least one prescription of ADHD-specific medication in the first year of follow-up			
General practitioner	123 of 759 (16%)	78 of 624 (13%)	–
Psych MD	526 of 759 (69%)	398 of 624 (64%)	–

One individual may have multiple ADHD diagnoses and prescriptions from multiple physicians.

Abbreviation: ADHD, attention-deficit/hyperactivity disorder.

a Tested outcomes: age and sex differences between individuals with initial ADHD-medication and no initial ADHD-medication. No statistical difference between individuals with initial ADHD-medication and without concerning sex (Chi-square test; *p* = 0.17) and age (*t* test; *p* = 0.42) at index.

Three-quarters of the study population were categorized as individuals without initial ADHD-specific medication (74%: 1,756 out of 2,380) and one-quarter was categorized as individuals with initial ADHD medication as they had received at least two prescriptions within the year of initial aADHD diagnosis (26%: 624 out of 2,380). Overall, about a third (32%: 759 out of 2,380) received at least one prescription of ADHD-specific medication in the year of initial aADHD diagnosis. ADHD-specific medication was prescribed mostly (69%: 526 out of 759) by the outpatient specialist, however, 16% (123 out of 759) of the individuals received a prescription by the GP.

### Psychiatric comorbidities before and after initial aADHD diagnosis

All individuals had at least one diagnosis of mental and behavioral disorders at index, including by definition aADHD (Table 2). After index, the number of individuals who continued to receive a documented aADHD diagnosis decreased from 100 to 50% in year two, 43% in year three, and 39% in year four. The percentage of individuals with any psychiatric diagnoses increased significantly from 50% 4 years before index to 100% at index (including aADHD) and decreased significantly 4 years after index to 72%. Individuals with initial ADHD medication showed significantly higher rates of psychiatric disorders than those without throughout the entire observation.

**Table 2.** Specific psychiatric comorbidities before and after initial aADHD diagnosis.

	*t* − 4	*t* − 3	*t* − 2	*t* − 1	*t* + 1	*t* + 2	*t* + 3	*t* + 4
Number of individuals observable before and after index (yearly cohorts)								
ADHD total (*N*)	535	1,032	1,648	2,380	2,380	1,845	1,348	732
Initial ADHD medication (*N*)	165	310	463	624	624	459	314	161
No initial ADHD medication (*N*)	370	722	1,185	1,756	1,756	1,386	1,034	571
Documentation of ADHD in study cohort throughout observation (no significance tested)								
At least one diagnosis of ADHD documented	–	–	–	–	2,380 (100%)	929 (50%)	574 (43%)	285 (39%)
Individuals with at least one diagnosis of mental and behavioral disorders (any)^a^								
ADHD total	269 (50%)	576 (56%)	978 (59%)	1,541 (65%)	2,380 (100%)	1,558 (79%)	997 (74%)	526 (72%)
Initial ADHD medication	91 (55%)	202 (65%)	301 (65%)	458 (73%)	624 (100%)	429 (93%)	273 (87%)	136 (84%)
No initial ADHD medication	178 (48%)	374 (52%)	677 (57%)	1,083 (62%)	1,756 (100%)	1,029 (74%)	724 (70%)	390 (68%)
Increase pre-index	*p* < 0.001; OR not calculated as every individual has ADHD in *t* + 1							
Decrease post-index	*p* < 0.001, OR not calculated as every individual has ADHD in *t* + 1							
No initial vs. initial ADHD medication	*p* < 0.001, OR not calculated as every individual has ADHD in *t* + 1							
Individuals with at least one diagnosis of mood disorder^a^								
ADHD total	170 (32%)	330 (32%)	565 (34%)	940 (39%)	1,265 (53%)	849 (46%)	584 (43%)	291 (40%)
Increase pre-index	*p* < 0.001; OR 2.17; 95% CI [1.91–2.48]							
Decrease post-index	*p* < 0.001; OR 0.75; 95% CI [0.66–0.85]							
Initial ADHD medication	64 (39%)	124 (40%)	213 (46%)	311 (50%)	405 (65%)	277 (60%)	177 (56%)	85 (53%)
Increase pre-index	*p* < 0.001; OR 2.17; 95% CI [1.68–2.80]							
Decrease post-index	*p* = 0.141; OR 0.82; 95% CI [0.64–1.06]							
No initial ADHD medication	106 (29%)	206 (29%)	352 (30%)	629 (36%)	860 (49%)	572 (41%)	407 (39%)	206 (36%)
Increase pre-index	*p* < 0.001; OR 2.27; 95% CI [1.94–2.66]							
Decrease post-index	*p* < 0.001; OR 0.73; 95% CI [0.63–0.85]							
No initial vs. initial ADHD medication	*p* < 0.001; OR 0.52; 95% CI [0.43–0.63]							

Different sample sizes during observation due to yearly cohorts—see “Methods.”

Abbreviations: ADHD, attention-deficit/hyperactivity disorder; CI, confidence intervals; OR, odds ratio.

a Outcome tested: (any) mental and behavioral disorders and mood disorder; Odds Ratios not calculated for (any) mental and behavioral disorders as every individual has at least one mental and behavioral disorder in *t* + 1 (ADHD). Significance tested based on Chi-square test; for time-wise comparison: increase *t* − 2 to t + 1 and decrease *t* + 1 to *t* + 2; for group comparison: initial ADHD-medication to no initial ADHD-medication compared at *t* + 1.

In the year of incident aADHD diagnosis, 56% of the individuals had documented anxiety disorders, 53% mood disorders, 20% SUD, and 19% personality and behavioral disorders (Supporting Information). For all these psychiatric comorbidities, an increase before and decrease after index was observed. However, for mood disorders in individuals with initial ADHD medication, the percentage of affected individuals was higher after index compared with before index.

### Therapies before and after initial aADHD diagnosis

In the year of initial aADHD diagnosis, 32% of individuals received at least one prescription of ADHD-specific medication, which decreased significantly to 13% within 4 years (Table 3). A significant decrease was also observed for those with initial ADHD-medication: 4 years after index, less than half still received medication (41%).

**Table 3.** Specific medication therapy and psychotherapy before and after initial aADHD diagnosis.

	*t* − 4	*t* − 3	*t* − 2	*t* − 1	*t* + 1	*t* + 2	*t* + 3	*t* + 4
Number of individuals observable before and after index (yearly cohorts)								
ADHD total (*N*)	535	1,032	1,648	2,380	2,380	1,845	1,348	732
Initial ADHD medication (*N*)	165	310	463	624	624	459	314	161
No initial ADHD medication (*N*)	370	722	1,185	1,756	1,756	1,386	1,034	571
Individuals with at least one ADHD-specific prescription^a^								
ADHD total	*–*	–	–	–	759 (32%)	358 (19%)	198 (15%)	98 (13%)
Decrease post-index			*p* < 0.001; OR 0.52; 95% CI [0.45–0.60]					
Initial ADHD medication	–	–	–	–	624 (100%)	293 (64%)	145 (46%)	66 (41%)
Decrease post-index			*p* < 0.001; OR 0.003 95% CI [0.00–0.02]					
Individuals with at least one antidepressants prescription^a^								
ADHD total	121 (23%)	234 (23%)	390 (24%)	656 (28%)	852 (36%)	511 (28%)	341 (25%)	183 (25%)
Increase pre-index			*p* < 0.001; OR 1.80; 95% CI [1.56–2.08]					
Decrease post-index			*p* < 0.001; OR 0.68; 95% CI [0.60–0.78]					
Initial ADHD medication	47 (28%)	87 (28%)	146 (32%)	235 (38%)	268 (43%)	152 (33%)	97 (31%)	54 (34%)
Increase pre-index			*p* < 0.001; OR 1.64; 95% CI [1.27–2.14]					
Decrease post-index			*p* = 0.001; OR 0.65; 95% CI [0.50–0.85]					
No initial ADHD medication	74 (20%)	147 (20%)	244 (21%)	421 (24%)	584 (33%)	359 (26%)	244 (24%)	129 (23%)
Increase pre-index			*p* < 0.001; OR 1.93; 95% CI [1.62–2.30]					
Decrease post-index			*p* < 0.001; OR 0.70; 95% CI [0.60–0.82]					
No initial vs. initial ADHD medication			*p* < 0.001; OR 0.66; 95% CI [0.54–0.80]					
Individuals with at least one psychotherapy session^a^								
ADHD total	187 (35%)	378 (37%)	627 (38%)	1,088 (46%)	1,483 (62%)	794 (43%)	509 (38%)	257 (35%)
Increase pre-index			*p* < 0.001; OR 2.70; 95% CI [2.36–3.07]					
Decrease post-index			*p* < 0.001; OR 0.46; 95% CI [0.40–0.52]					
Initial ADHD medication	63 (38%)	133 (43%)	203 (44%)	350 (56%)	466 (75%)	245 (53%)	140 (45%)	69 (43%)
Increase pre-index			*p* < 0.001; OR 3.77; 95% CI [2.90–4.93]					
Decrease post-index			*p* < 0.001; OR 0.39; 95% CI [0.30–0.51]					
No initial ADHD medication	124 (34%)	245 (34%)	424 (36%)	738 (42%)	1,017 (58%)	549 (40%)	369 (36%)	188 (33%)
Increase pre-index			*p* < 0.001; OR 2.47; 95% CI [2.12–2.88]					
Decrease post-index			*p* < 0.001; OR 0.48; 95% CI [0.41–0.55]					
No initial vs. initial ADHD medication			*p* < 0.001; OR 0.47; 95% CI [0.38–0.57]					
Individuals with at least one visit at the Psych MD (no significance tested)								
ADHD total	119 (22%)	238 (23%)	384 (23%)	731 (31%)	1,326 (56%)	667 (37%)	453 (34%)	221 (30%)
Initial ADHD medication	51 (31%)	90 (29%)	149 (32%)	255 (41%)	510 (82%)	271 (59%)	154 (49%)	72 (45%)
No initial ADHD medication	68 (18%)	148 (20%)	235 (20%)	476 (27%)	816 (46%)	406 (29%)	299 (29%)	149 (26%)
Individuals with at least one visit at the Psych (no significance tested)								
ADHD total	49 (9%)	88 (9%)	176 (11%)	308 (13%)	467 (20%)	277 (15%)	166 (12%)	68 (9%)
Initial ADHD medication	23 (14%)	42 (14%)	70 (15%)	108 (17%)	161 (26%)	100 (22%)	54 (17%)	22 (14%)
No initial ADHD medication	26 (7%)	46 (6%)	106 (9%)	200 (11%)	306 (17%)	177 (13%)	112 (11%)	46 (8%)

Different sample sizes during observation due to yearly cohorts—see “Methods.”

Abbreviations: ADHD, attention-deficit/hyperactivity disorder; CI, confidence intervals; OR, odds ratio.

a Significance tested based on Chi-square test; for time-wise comparison: increase *t* − 2 to *t* + 1 and decrease *t* + 1 to *t* + 2; for group comparison: initial ADHD-medication to no initial ADHD-medication compared at *t* + 1.

In the total ADHD cohort, the most common co-medication was antidepressants (36% of individuals in *t* + 1). Before index, a significant increase and thereafter a decrease was observed. However, more individuals continued to receive a prescription after index compared with the time period before index. Throughout observation, the percentage of individuals receiving antidepressants was significantly higher in the subgroup “initial ADHD-medication” compared to the subgroup “no initial ADHD medication.” For all other classes of co-medication analyzed, a prescription peak was observed in the year of the index: 10% of individuals received antipsychotics, 6% hypnotics and sedatives, and 4% anxiolytics. Medication for SUD was not reported due to data protection (*n* < 5).

Sixty-two percent of individuals received psychotherapy at *t* + 1, and around 30% in the years before and after index. Again, individuals with initial ADHD medication showed higher percentages throughout observation with a peak at index (initial ADHD medication: 75%; no initial ADHD medication 58%). 20–30% of the individuals with psychotherapy only had probatory sessions (supplement). Around three-quarters of the individuals consulted a specialist in the year of aADHD diagnosis (56% Psych MD, 20% Psych). The percentage was higher for individuals who received initial ADHD medication than those who did not (Psych MD 82% vs. 26%, Psych: 46% vs. 17%;). The number of individuals increased before index and decreased thereafter; however, more individuals consulted specialists after index than before. Independent of the initial medication regime, around 90% of the individuals attended the GP throughout the entire observational period (supplement). Hospitalizations, also due to psychiatric reasons, peaked at index and decreased thereafter, however, increased again in *t* + 4 for those with initial ADHD-medication (supplement).

### Healthcare costs before and after initial aADHD diagnosis

In t + 1, healthcare costs were, on average, €4,006 per individual (Supporting Information). In the years before index, an increase in costs was observed, peaking at *t* + 1, but decreased again afterward, reaching a level similar to the years before diagnosis. Main cost drivers at index were psychotherapy (€2,772), inpatient (€1,747), and outpatient care (€1,276). While most costs after index decreased to a similar level as before, the costs for psychotherapy were around twice as high 4 years after index compared with 4 years before index.

For individuals with and without initial ADHD medication, the costs peaked at *t* + 1 (€5.442 and €3.485), with a significant increase before diagnosis and decrease thereafter, Table 4. For those with initial ADHD medication, the costs increased again in *t* + 4. In both subgroups, the main cost drivers were similar to the overall cohort. Especially, psychotherapy was a relevant cost factor for individuals with initial ADHD medication even before index. However, costs were notably higher throughout all years of observation for all cost categories and for individuals with initial ADHD medication compared to those without.

**Table 4.** Direct healthcare costs [€], sickness benefits [€], and sick leave days [days] before and after initial aADHD diagnosis—comparison of cohorts with initial and without initial ADHD medication.

	*t* − 4	*t* − 3	*t* − 2	*t* − 1	*t* + 1	*t* + 2	*t* + 3	*t* + 4
Initial ADHD medication (mean ± SD)								
Total healthcare costs^a^	2,647 ± 5,234	2,803 ± 4,494	2,775 ± 4,475	3,951 ± 6,099	5,442 ± 6,195	3,818 ± 4,375	3,514 ± 4,750	4,144 ± 6,137
Inpatient care	1,233 ± 4,624	1,481 ± 4,687	1,072 ± 3,457	1,493 ± 4,415	1,884 ± 4,777	1,231 ± 4,229	1,414 ± 4,890	1,940 ± 5,833
Outpatient care	732 ± 823	886 ± 881	897 ± 882	1,182 ± 1,161	1,932 ± 1,662	1,565 ± 1,449	1,221 ± 1,148	1,054 ± 1,011
Psychotherapy^a^	1,350 ± 3,649	1,791 ± 5,276	1,167 ± 3,034	2,172 ± 5,189	4,580 ± 9,750	3,927 ± 9,503	2,509 ± 7,392	1,727 ± 5,229
Aids and remedies	87 ± 216	117 ± 265	114 ± 273	135 ± 329	198 ± 485	195 ± 497	177 ± 449	171 ± 392
Medication	222 ± 395	225 ± 381	272 ± 460	300 ± 515	827 ± 771	658 ± 745	564 ± 732	541 ± 744
Sickness benefit	388 ± 2,524	336 ± 2,628	406 ± 2,737	878 ± 4,222	483 ± 2,456	225 ± 1,600	553 ± 3,875	515 ± 3,606
Sick leave days (days)	21 ± 99	18 ± 58	23 ± 85	39 ± 114	21 ± 71	18 ± 55	19 ± 71	21 ± 76
No initial ADHD medication (mean ± SD)								
Total healthcare costs^a^	1,599 ± 2,508	1,550 ± 2,294	1,941 ± 3,152	2,142 ± 3,510	3,485 ± 5,001	2,323 ± 3,473	2,108 ± 3,098	1,785 ± 2,732
Inpatient care	890 ± 3,111	706 ± 2,561	916 ± 3,005	884 ± 3,073	1,700 ± 4,472	957 ± 3,053	863 ± 2,547	746 ± 2,856
Outpatient care	550 ± 591	584 ± 608	637 ± 672	735 ± 795	1,072 ± 990	839 ± 917	769 ± 821	673 ± 758
Psychotherapy^a^	597 ± 2,129	512 ± 1,742	942 ± 3,347	1,400 ± 4,425	2,234 ± 5,658	1,727 ± 5,807	1,587 ± 5,707	1,266 ± 4,847
Aids and remedies	83 ± 219	94 ± 247	95 ± 231	96 ± 257	122 ± 287	110 ± 259	111 ± 271	128 ± 380
Medication	192 ± 420	178 ± 381	165 ± 331	169 ± 315	233 ± 382	220 ± 399	211 ± 376	195 ± 360
Sickness benefit	163 ± 1,306	119 ± 1,084	246 ± 1,604	370 ± 2,316	475 ± 2,505	251 ± 1,776	210 ± 2,041	220 ± 2,004
Sick leave days (days)	13 ± 43	13 ± 48	17 ± 59	20 ± 66	20 ± 64	15 ± 49	13 ± 44	15 ± 55

Abbreviations: ADHD, attention-deficit/hyperactivity disorder; SD, standard deviation.

a Outcomes tested were: total healthcare costs and costs for psychotherapy. Significance tested based on Wilcoxon test; increase *t* − 2 to *t* + 1 and decrease *t* + 1 to *t* + 2; for group comparison: “initial ADHD-medication” to no “initial ADHD-medication” compared at *t* + 1; all tests were significant at a level of *p* < 0.001.

Sickness benefits and sick leave days were notably elevated for all incident aADHD individuals in the year of initial aADHD diagnosis, peaking already in *t* − 1. They were especially elevated for those with initial ADHD medication (*t* − 1 39 days and €878; *t* + 1 21 days and €483).

## Discussion

### Key findings

In this statutory health claims data analysis, we described disease burden, treatment allocation, and healthcare costs for 2,380 individuals over 4 years before and after the initial aADHD diagnosis.

In the years before aADHD diagnosis, psychiatric comorbidities, disorder-specific medication (primarily antidepressants), and psychotherapy increased, peaking in the year of diagnosis. A similar pattern was observed for healthcare costs. However, the number of sick leave days and sickness benefits peaked in the year before diagnosis.

Most individuals received diagnosis from a specialist: 23% in the inpatient setting and another 33% by the outpatient specialist (Psych MD); 41% were diagnosed by the GP. In the year of diagnosis, 76% of individuals had a consultation with a specialist for CNS disorders, decreasing to 39% in *t* + 4. Over the years following initial diagnosis, the prevalence of psychiatric comorbidities and prescriptions of disorder-specific drugs, especially antidepressants, psychotherapy, and healthcare costs decreased again but were higher compared with levels observed in the years before diagnosis. Moreover, the proportion of individuals who continued to receive an aADHD diagnosis decreased to 39% in *t* + 4.

In the year of initial diagnosis, 32% of individuals received at least one, and 26% at least two prescriptions of ADHD-specific medication. Specialists for CNS disorders (psychiatrists) prescribed ADHD-specific medication in most cases (69%). Four years after diagnosis, only 13% of individuals received ADHD-specific medication.

Throughout observation, comorbidities, corresponding medication, psychotherapy, healthcare costs, sickness benefit, and sick leave days were more frequent in individuals with initial ADHD medication than those without, suggesting a higher disease burden. Healthcare costs, costs for inpatient care, sick leave days, and sickness benefits in this subgroup initially decreased in the first 2 years following diagnosis of aADHD but tended to increase again in *t* + 3 and *t* + 4.

### Interpretation of findings

ADHD is a neurodevelopmental disorder beginning in childhood or early adolescence. It often persists into adulthood with different trajectories over the lifespan [15]. It has been suggested that cases of “late-onset ADHD” might exist (>12 years) but are probably restricted to only few cases [1,16]. Thus, we did not expect to find a rather advanced average age of 35 years in our dataset.

ADHD has long been considered a “childhood disorder,” and recent findings suggest that there is still a significant lack of consensus among psychiatrists and other physicians with respect to core symptoms and valid diagnostic procedures for aADHD, leaving the disorder underdiagnosed [2,17]. Here, less than 50% of individuals had a consultation with a psychiatrist or psychologist in the years before the initial aADHD diagnosis. Accordingly, aADHD was possibly not recognized or misdiagnosed over a prolonged period in a relevant proportion of individuals, especially as comorbidities may mask ADHD symptoms, complicate, and delay diagnosis [6,18,19].

Increasing comorbidities and use of disorder-specific medication, psychotherapy, and healthcare costs suggest a steadily increasing symptom burden in individuals over the years before aADHD is diagnosed. It has been shown that a time delay in diagnosis of aADHD may lead to an onset or deterioration of various psychiatric comorbidities [20–22]. Assuming that in the sample investigated here the underlying ADHD condition was present but not recognized, we suggest that disease burden increased over the years as a result of inappropriate or insufficient interventions with respect to the underlying condition.

In line with previous investigations into the healthcare situation for adults with ADHD [5], the GP plays an important role as the diagnosing physician, as 41% of individuals were initially diagnosed by a GP. However, ADHD-specific medication was primarily prescribed by psychiatrists or in the inpatient setting.

An important finding of the present investigation is that in the year of initial diagnosis, less than one-third of individuals received any prescription of ADHD-specific medication, and only a fourth got at least two prescriptions. At the same time, almost two-thirds of individuals received psychotherapy in the year of aADHD diagnosis. In about one-third of individuals, psychotherapy had been initiated already before diagnosis of aADHD, presumably for treatment of comorbid disorders. Guidelines in practice during the observational period of the present investigation recommended a dual treatment approach with stimulant medication and psychotherapy as the first choice [23]. In current German guidelines, ADHD-specific medication is recommended as the first choice also in mild and moderate cases of aADHD, along with psychoeducation [14]. Apparently, despite the vast body of evidence for the beneficial effects of ADHD-specific medication, at least in the short-term [24], and in contrast to the recommendation in previous and current guidelines, stimulant medication is only reluctantly prescribed in newly identified adults with ADHD. Moreover, significantly higher comorbidities, healthcare costs, and sick leave days in individuals with initial ADHD medication compared to those without suggest that medication is preferentially initiated in individuals with a pronounced and heavier disease burden.

After aADHD diagnosis, psychiatric comorbidities, medication use, utilization of psychotherapy, and total healthcare costs generally decreased to levels observed in the years before diagnosis. Only half of the individuals still received a clinical diagnosis of aADHD at *t* + 2, and counts continued to decrease. A similar trend was recorded for prescriptions of ADHD-specific medication. These trends may indicate some improvement of the psychiatric conditions in individuals following diagnosis and initiation of treatment for aADHD. Thus, it is possible that some individuals did not wish or require to receive further treatment.

However, in patients with a higher disease burden, healthcare costs, costs for inpatient care, and sick leave days tended to increase again in *t* + 3 and *t* + 4, indicating a deterioration of the psychiatric conditions. Almost all individuals had a consultation with an expert of CNS disorders in the year of diagnosis, but this proportion steadily decreased to only about 60% in *t* + 4. At the same time, less than half still received a prescription of ADHD-specific medication at year four. The intensity of healthcare utilization decreased in this subgroup, which may have compromised more favorable outcomes over a prolonged period.

Interestingly, healthcare utilization in the years after aADHD diagnosis decreased considerably for the population investigated here, and healthcare costs, comorbidities, medication use, and disease burden as indicated by sick leave days generally returned to but did not fall below pre-diagnostic levels. This, however, would be expected if treatment initiated after aADHD diagnosis did not merely lead to a transient mitigation of a slowly deteriorating situation, but to a substantial improvement of the overall psychiatric conditions of individuals. Thus, a relevant proportion of individuals may not be satisfied with the care they receive in the outpatient setting, stop making appointments with the treating physician, and drop out of the medical system, even though the medical condition would necessitate continuation of care.

In a large clinical trial, initiation of a comprehensive, multimodal ADHD-specific treatment program over 1 year has been shown to effectively reduce ADHD core symptoms and improve functionality in aADHD [25]. These beneficial effects were maintained over another 1.5 years following the controlled phase of the study, demonstrating that treatment according to guidelines for at least 1 year can be very effective in the long-term [26].

Overall, our findings suggest that significant deficiencies may currently exist with respect to the reality of healthcare for aADHD in Germany, and that guideline recommendations are not yet comprehensively implemented in everyday routine care. We argue that lack of experience with aADHD may represent one explanation, highlighting the necessity for more educational programs on this disorder for experts of CNS disorders and GPs. GPs apparently are a major initial contact point and conduct in many cases an initial clinical evaluation of psychiatric conditions. Accordingly, medical education, and a close cooperation between specialities is essential.

We suggest that the establishment of regional centers specializing in the management of aADHD may represent a promising measure to improve the healthcare situation in the outpatient setting. These centers would act as a central point of contact for physicians and individuals with aADHD, offering physicians the opportunity to refer their individuals directly to ADHD specialists. There, individuals could be evaluated and, upon confirmation of the diagnosis, specific treatment measures would be initiated according to guideline recommendations. Avoiding a delay of the diagnosis and initiating a multimodal treatment program, tailored to the individual needs, could reduce escalation of the disease burden, and consequently healthcare costs [27,28]. In addition, these centers can act as a supervisory body overseeing, managing, and improving the reality of care through intersectoral collaboration while also saving resources within the healthcare system.

### Strengths and limitations

This study’s major strength is the longitudinal design, that is the availability of data covering 4 years before and 4 years after the initial diagnosis of aADHD. In addition, the multitude of available endpoints within this representative sample of the German population [13] provides valuable insights into the reality of care and costs as experienced by individuals with aADHD.

In this study, we set out to analyze adults with initial ADHD, who did not show prevalent diagnosis at least 365 days prior to the initial adult ADHD diagnosis. By doing so, we excluded individuals with continuous diagnoses of ADHD throughout early adulthood. This may bias our cohort toward individuals who are not within optimal care throughout early adulthood [4,6]. In addition, only documented aADHD cases could be analyzed, which may bias our study population toward individuals with more severe symptoms of aADHD or with psychiatric comorbidities seeking out professional help. Furthermore, individuals may have received diagnosis of ADHD in childhood, which could not be analyzed within the 6-year timeframe of our study. This study is based on SHI claims data, which are recorded for the primary purpose of billing. Thus, this source of data is limited in terms of primary information by physicians and individuals themselves and does not depict costs when paid out-of-pocket.

## Conclusion

We conclude that adult individuals with ADHD in the current German healthcare system may be recognized too late, misdiagnosed due to comorbid conditions masking ADHD symptoms, or only diagnosed rather late in mid-adulthood, even though disease burden is high. Following diagnosis, most individuals do not receive continuous professional medical care for time periods exceeding 1 year. Interventions, especially ADHD-specific medication, are not initiated as recommended in guidelines, and the overall effectiveness of routine clinical care to ameliorate disease burden associated with aADHD is limited. Regional centers specializing in ADHD could provide a central point of contact for outpatient physicians and individuals, which could be an effective measure to improve the reality of care.

## Data Availability

Due to the sensitivity of the data and data protection regulations, the analysis datasets of the current study will not be shared or stored at a public repository. Analysis datasets can be assessed upon request at the Institute for Applied Health Research Berlin (InGef) (info@ingef.de), if required.

## Supplementary material

For supplementary material accompanying this paper visit https://doi.org/10.1192/j.eurpsy.2020.84. click here to view supplementary material
