# Supplementary material for: Disease burden and direct medical costs of incident adult ADHD: A retrospective longitudinal analysis based on German statutory health insurance claims data
Source: Eur Psychiatry. 2020 Oct 1;63(1):e86. doi: 10.1192/j.eurpsy.2020.84 (PMC7576526; doi:10.1192/j.eurpsy.2020.84)
Supplement: Supplementary file 1 [file S092493382000084Xsup001.docx]

# SUPPLEMENT

### Table 5: specific psychiatric comorbidities, therapies and resource use before and after initial aADHD diagnosis

|  | **t-4** | **t-3** | **t-2** | **t-1** | **t+1** | **t+2** | **t+3** | **t+4** |
| --- | --- | --- | --- | --- | --- | --- | --- | --- |
| **Number of individuals observable before and after index (yearly cohorts)** | | | | | | | | |
| ADHD total (**N**) | 535 | 1,032 | 1,648 | 2,380 | 2,380 | 1,845 | 1,348 | 732 |
| Initial ADHD- medication (**N**) | 165 | 310 | 463 | 624 | 624 | 459 | 314 | 161 |
| No initial ADHD-medication (**N**) | 370 | 722 | 1,185 | 1,756 | 1,756 | 1,386 | 1,034 | 571 |
| Individuals with at least one diagnosis of **substance use disorder (SUD)** | | | | | | | | |
| ADHD total | 54 (10%) | 125 (12%) | 216 (13%) | 352 (15%) | 467 (20%) | 312 (17%) | 193 (14%) | 100 (14%) |
| Initial ADHD-medication | 15 (9%) | 43 (14%) | 56 (12%) | 101 (16%) | 127 (20%) | 77 (17%) | 49 (16%) | 27 (17%) |
| No initial ADHD-medication | 39 (11%) | 82 (11%) | 160 (14%) | 251 (14%) | 340 (19%) | 235 (17%) | 144 (14%) | 73 (13%) |
| Individuals with at least one diagnosis of **anxiety disorder** | | | | | | | | |
| ADHD total | 173 (32%) | 387 (38%) | 663 (40%) | 1,057 (44%) | 1,325 (56%) | 829 (45%) | 565 (42%) | 282 (39%) |
| Initial ADHD-medication | 60 (36%) | 142 (46%) | 204 (44%) | 332 (53%) | 380 (61%) | 239 (52%) | 143 (46%) | 70 (43%) |
| No initial ADHD-medication | 113 (31%) | 245 (34%) | 459 (39%) | 725 (41%) | 945 (54%) | 590 (43%) | 422 (41%) | 212 (37%) |
| Individuals with at least one diagnosis of **personality and behavioural disorders** | | | | | | | | |
| ADHD total | 43 (8%) | 110 (11%) | 164 (10%) | 281 (12%) | 455 (19%) | 269 (15%) | 184 (14%) | 86 (12%) |
| Initial ADHD-medication | 17 (10%) | 42 (14%) | 55 (12%) | 87 (14%) | 125 (20%) | 77 (17%) | 51 (16%) | 21 (13%) |
| No initial ADHD-medication | 26 (7%) | 68 (9%) | 109 (9%) | 194 (11%) | 330 (19%) | 192 (14%) | 133 (13%) | 65 (11%) |
| Individuals with at least one **antipsychotics** prescription | | | | | | | | |
| ADHD total | 39 (7%) | 67  (6%) | 112 (7%) | 173 (7%) | 248 (10%) | 146 (8%) | 92  (7%) | 59  (8%) |
| Initial ADHD-medication | 16 (10%) | 24  (8%) | 36  (8%) | 51  (8%) | 75 (12%) | 36  (8%) | 25  (8%) | 20 (12%) |
| No initial ADHD- medication | 23 (6%) | 43  (6%) | 76  (6%) | 122 (7%) | 173 (10%) | 110 (8%) | 67 (6%) | 39  (7%) |
| Individuals with at least one **anxiolytics** prescription | | | | | | | | |
| ADHD total | 18 (3%) | 32  (3%) | 48  (3%) | 117 (15%) | 96  (4%) | 71  (4%) | 41  (3%) | 21  (3%) |
| Initial ADHD-medication | 10 (6%) | 11  (4%) | 20  (4%) | 46  (7%) | 29  (5%) | 28  (6%) | 13  (4%) | 6  (4%) |
| No initial ADHD-medication | 8 (2%) | 21  (3%) | 28  (2%) | 71  (4%) | 67  (4%) | 43  (3%) | 28  (3%) | 15  (3%) |
| Individuals with at least one **hypnotics and sedatives** prescription | | | | | | | | |
| ADHD total | 18 (3%) | 32  (3%) | 66  (4%) | 112 (5%) | 140 (6%) | 84  (5%) | 47  (3%) | 31  (4%) |
| Initial ADHD-medication | 5 (3%) | 9  (3%) | 21  (5%) | 36  (6%) | 48  (8%) | 24  (5%) | 11  (4%) | 9  (6%) |
| No initial ADHD-medication | 13 (4%) | 23  (3%) | 45  (4%) | 76  (4%) | 92  (5%) | 60  (4%) | 36  (3%) | 22  (4%) |
| Individuals with **only probatory sessions** of those with at least one psychotherapy session | | | | | | | | |
| ADHD total | 39 (21%) | 75 (20%) | 139 (22%) | 298 (27%) | 432 (29%) | 152 (19%) | 99 (19%) | 45 (18%) |
| Initial ADHD-medication | 17 (27%) | 30 (23%) | 50 (25%) | 102 (29%) | 150 (32%) | 55 (22%) | 22 (16%) | 18 (26%) |
| No initial ADHD-medication | 22 (18%) | 45 (18%) | 89 (21%) | 196 (27%) | 282 (28%) | 97 (18%) | 77 (21%) | 27 (14%) |
| Individuals with at least one visit at the **GP** | | | | | | | | |
| ADHD total | 476 (89%) | 925 (90%) | 1,468 (89%) | 2,114 (89%) | 2,281 (96%) | 1,668 (90%) | 1,210 (90%) | 661 (90%) |
| Initial ADHD-medication | 151 (92%) | 284 (92%) | 422 (91%) | 578 (93%) | 593 (95%) | 423 (92%) | 283 (90%) | 153 (95%) |
| No initial ADHD-medication | 325 (88%) | 641 (89%) | 1,046 (88%) | 1,536 (87%) | 1,688 (96%) | 1,245 (90%) | 927 (90%) | 508 (89%) |
| Individuals with at least one **hospitalisation** | | | | | | | | |
| ADHD total | 89 (17%) | 191 (19%) | 311 (19%) | 444 (19%) | 623 (26%) | 347 (19%) | 262 (19%) | 116 (16%) |
| Initial ADHD-medication | 22 (13%) | 64 (21%) | 88 (19%) | 127 (20%) | 166 (27%) | 91 (20%) | 54 (17%) | 41 (25%) |
| No initial ADHD-medication | 67 (18%) | 127 (18%) | 223 (19%) | 317 (18%) | 457 (26%) | 256 (18%) | 208 (20%) | 75 (13%) |
| Individuals with at least one **hospitalisation due to psychiatric problems** (primary diagnosis ICD-10 F) * | | | | | | | | |
| ADHD total | 22 (4%) | 51  (5%) | 90  (5%) | 134 (6%) | 288 (12%) | 120 (7%) | 83  (6%) | 37  (5%) |
| Increase pre-index | P<0.001; OR 4.42; 95% CI [3.67 – 5.34] | | | | | | | |
| Decrease post-index | P<0.001; OR 0.46; 95% CI [0.40 – 0.54] | | | | | | | |
| Initial ADHD-medication | 8 (5%) | 23  (7%) | 24  (5%) | 41  (7%) | 76 (12%) | 32  (7%) | 15  (5%) | 12  (7%) |
| Increase pre-index | P<0.001; OR 6.17; 95% CI [4.29 – 9.04] | | | | | | | |
| Decrease post-index | P<0.001; OR 0.57; 95% CI [0.43 – 0.74] | | | | | | | |
| No ADHD-initial medication | 14 (4%) | 28  (4%) | 66  (6%) | 93  (5%) | 212 (12%) | 88  (6%) | 68  (7%) | 25  (4%) |
| Increase pre-index | P<0.001; OR 3.90; 95% CI [3.14 – 4.87] | | | | | | | |
| Decrease post-index | P<0.001; OR 0.43; 95% CI [0.35 – 0.51] | | | | | | | |
| No initial vs. initial ADHD-medication | P<0.001; OR 0.71; 95% CI [0.59 – 0.87] | | | | | | | |

** Significance tested based on Chi² test; for time-wise comparison: increase t-2 to t+1 and decrease t+1 to t+2; for group comparison: ‘initial ADHD-medication’ to no ‘initial ADHD-medication’ compared at t+1*

# SUPPLEMENT

### Table 6: Direct healthcare costs [€], sickness benefits [€] and sick leave days [days] before and after initial aADHD diagnosis – total ADHD population

|  | **t-4** | **t-3** | **t-2** | **t-1** | **t+1** | **t+2** | **t+3** | **t+4** |
| --- | --- | --- | --- | --- | --- | --- | --- | --- |
| **ADHD total** (mean ± SD) | | | | | | | | |
| **Total Healthcare costs*** | 1,986  ± 3,640 | 1,892  ± 2,914 | 2,131  ± 3,386 | 2,613  ± 4,301 | 4,006  ± 5,411 | 2,693  ± 3,749 | 2,389  ± 3,394 | 2,230  ± 3,423 |
| Inpatient care | 1,027  ± 3,826 | 940  ± 3,341 | 967  ± 3,185 | 1,061  ± 3,592 | 1,747  ± 4,549 | 1,001  ± 3,195 | 962  ± 2,954 | 1,005  ± 3,689 |
| Outpatient care | 608  ± 678 | 679  ± 720 | 706  ± 731 | 846  ± 898 | 1,276  ± 1,172 | 1,013  ± 1,083 | 878  ± 931 | 761  ± 842 |
| Psycho-therapy* | 833  ± 2,699 | 924  ± 3,249 | 997  ± 3,206 | 1,600  ± 4,637 | 2,772  ± 6,630 | 2,269  ± 6,912 | 1,795  ± 6,113 | 1,359  ± 4,883 |
| Aids and remedies | 84  ± 219 | 101  ± 254 | 102  ± 252 | 108  ± 285 | 141  ± 342 | 134  ± 339 | 124  ± 310 | 140  ± 391 |
| Medication | 199  ± 403 | 190  ± 373 | 194  ± 371 | 199  ± 359 | 386  ± 547 | 331  ± 538 | 287  ± 470 | 288  ± 537 |
| Sickness benefit | 229  ± 1,720 | 173  ± 1,488 | 282  ± 1,870 | 517  ± 3,071 | 476  ± 2,401 | 246  ± 1,746 | 292  ± 2,610 | 288  ± 2,488 |
| Sick leave days [days] | 16  ± 66 | 15  ± 51 | 19  ± 67 | 25  ± 82 | 21  ± 66 | 16  ± 50 | 14  ± 50 | 16  ± 60 |

*Outcomes tested were: total healthcare costs and costs for psychotherapy. Significance tested based on Wilcoxon test; increase t-2 to t+1 and decrease t+1 to t+2; for group comparison: ‘initial ADHD-medication’ to no ‘initial ADHD-medication’ compared at t+1; all tests were significant at a level of p<0.001
